# High-resolution 3D volumetry versus conventional measuring techniques for the assessment of experimental lymphedema in the mouse hindlimb

**DOI:** 10.1038/srep34673

**Published:** 2016-10-04

**Authors:** Florian S. Frueh, Christina Körbel, Laura Gassert, Andreas Müller, Epameinondas Gousopoulos, Nicole Lindenblatt, Pietro Giovanoli, Matthias W. Laschke, Michael D. Menger

**Affiliations:** 1 Institute for Clinical and Experimental Surgery, Saarland University, 66421 Homburg/Saar, Germany; 2 Division of Plastic Surgery and Hand Surgery, University Hospital Zurich, 8091 Zurich, Switzerland; 3 Clinic of Diagnostic and Interventional Radiology, Saarland University Medical Center, Homburg/Saar, Germany; 4 Institute of Pharmaceutical Sciences, Swiss Federal Institute of Technology, ETH Zurich, Zurich, Switzerland

## Abstract

Secondary lymphedema is a common complication of cancer treatment characterized by chronic limb swelling with interstitial inflammation. The rodent hindlimb is a widely used model for the evaluation of novel lymphedema treatments. However, the assessment of limb volume in small animals is challenging. Recently, high-resolution three-dimensional (3D) imaging modalities have been introduced for rodent limb volumetry. In the present study we evaluated the validity of microcomputed tomography (μCT), magnetic resonance imaging (MRI) and ultrasound in comparison to conventional measuring techniques. For this purpose, acute lymphedema was induced in the mouse hindlimb by a modified popliteal lymphadenectomy. The 4-week course of this type of lymphedema was first assessed in 6 animals. In additional 12 animals, limb volumes were analyzed by μCT, 9.4 T MRI and 30 MHz ultrasound as well as by planimetry, circumferential length and paw thickness measurements. Interobserver correlation was high for all modalities, in particular for μCT analysis (r = 0.975, p < 0.001). Importantly, caliper-measured paw thickness correlated well with μCT (r = 0.861), MRI (r = 0.821) and ultrasound (r = 0.800). Because the assessment of paw thickness represents a time- and cost-effective approach, it may be ideally suited for the quantification of rodent hindlimb lymphedema.

The lymphatic system is important for the regulation of fundamental biological processes such as immune response, intestinal lipid absorption and tissue fluid homeostasis^1^,^2^. The cardinal manifestation of lymphatic dysfunction is lymphedema, a condition which is characterized by limb swelling, chronic interstitial inflammation and connective or fat tissue deposition^3^,^4^. Based on the triggering cause it can be classified into primary and secondary lymphedema^5^.

Primary lymphedema has a genetic background with a dysfunctional lymphatic system, either already symptomatic after birth or later in life^6^. In contrast, acquired damage of collecting lymphatic vessels causes secondary lymphedema. In the United States, more than 5 million people suffer from cancer-related lymphedema^7^, the most common form in developed countries. Lymph node dissection and irradiation result in the formation of scar tissue, which is a key inhibitor of lymphatic regeneration^8^. In particular, breast cancer and melanoma are associated with high rates of secondary lymphedema^9^. Despite reduced surgical invasiveness, recent data indicate that 20% of female breast cancer patients undergoing axillary lymph node dissection will develop arm lymphedema^10^. Given the livelong course of the disease, lymphedema has a highly relevant socio-economic burden.

Many animal models have been established for the analysis of lymphedema pathophysiology and the development of novel approaches for its treatment. However, the induction of chronic lymphedema in animals is challenging and requires lymphatic interruption by means of surgery and irradiation^11^. The rodent limb is a frequently used model to study lymphatic regeneration after lymph node resection^12^,^13^ and emerging lymphedema treatments such as vascularized lymph node transfer^14^,^15^,^16^,^17^,^18^ or stem cell transplantation^19^,^20^. For this purpose, the valid assessment of limb volume is a major prerequisite.

Recently, high-resolution three-dimensional (3D) imaging techniques such as magnetic resonance imaging (MRI) or microcomputed tomography (μCT) have been introduced for rodent limb volumetry^21^,^22^. However, the validity and reliability of these complex 3D techniques for limb volume assessment compared to conventional measuring techniques have not been systematically evaluated so far. Therefore, in the present study we assessed the hindlimb volumes in an acute lymphedema mouse model by means of μCT, 9.4 T MRI and high-resolution 30 MHz ultrasound (hrUS) as well as planimetry, circumferential length and paw thickness measurements. Subsequently, we calculated interobserver variability and performed a correlation analysis for the comparison of the different techniques.

## Results

### Acute hindlimb lymphedema model

**Figure 1:**
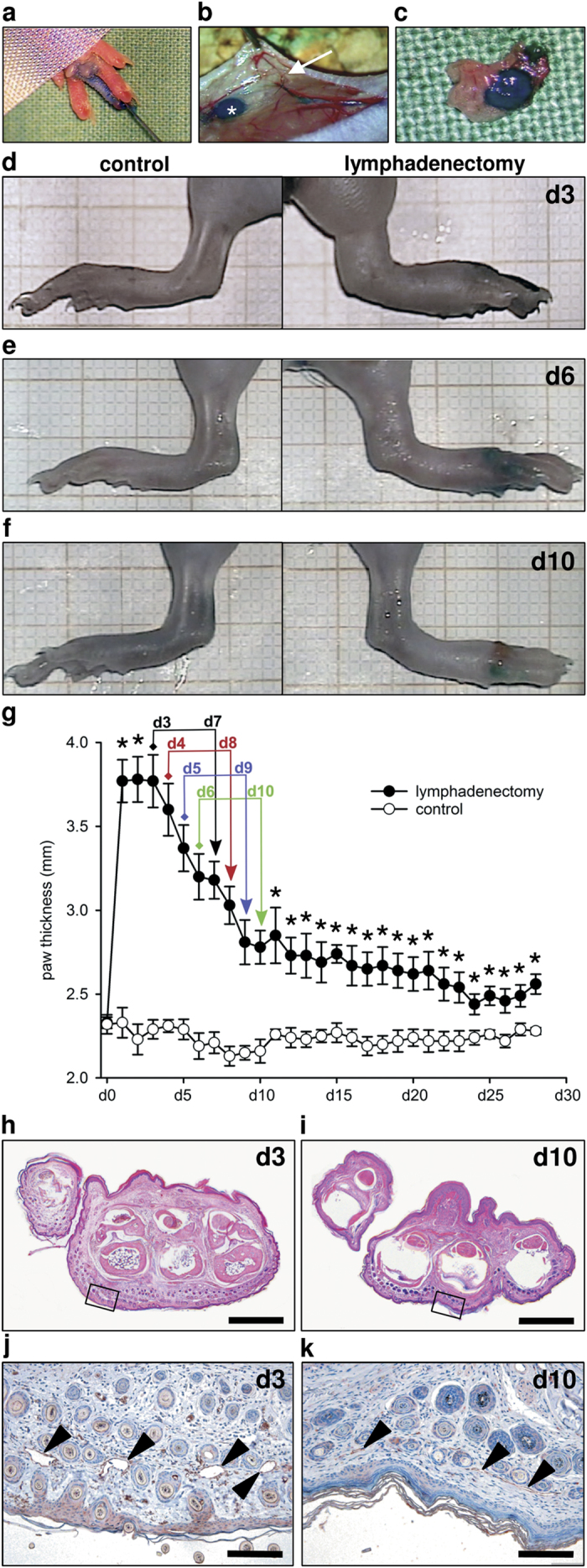
Animal model. (**a–c**) After methylene blue injection (**a**) the popliteal lymph node was visualized (**b**, asterisk). The afferent lymphatic vessels ran parallel to the iscial vein and were ligated (**b**, arrow). Subsequently, the popliteal fat pad including lymph nodes and efferent lymphatic vessels was resected (**c**). (**d–f**) Stereomicroscopic images illustrating paw edema with regression between day 3 (**d**) and day 10 (**f**). (**g**) Acute lymphedema in the mouse hindlimb over 28 days. Four experimental groups (n = 3 per group) were measured twice during the phase of postsurgical swelling (colored arrows) using different volumetric techniques. At the end of the experiment, there was still significant paw swelling (n = 6; mean ± SEM; *p < 0.05). (**h,i**) HE-stained paw cross-sections with increased dermal thickness 3 days (**h**) after lymph node dissection. Ten days after surgery (**i**), the paw volume had markedly decreased. Scales = 1 mm. (**j,k**) Inserts of (**h**,**i)**. Dermal lymphatic vessels were dilated on day 3 (**j**, arrowheads), but exhibited normal configuration on day 10 (**k**, arrowheads) as shown by means of immunohistochemical staining with LYVE-1. Scales = 140 μm.

Acute lymphedema was induced in C57BL/6 mice by means of popliteal lymphadenectomy, circular skin incision and cautery (Fig. 1a–c). In pilot experiments, we first assessed the course of this type of acute lymphedema during 28 days. Limb swelling peaked 1 to 3 days after surgery (maximal ratio operated/non-operated leg: 1.7) and rapidly decreased throughout the following observation period (Fig. 1d–f). Noteworthy, there was still significant paw swelling after 28 days (ratio: 1.1, p < 0.05) (Fig. 1g). Based on these results the evaluation of different volumetric techniques was performed in groups of 3 animals between day 3 and 10 after surgery, which guaranteed a well-distributed data set for correlation analyses (Fig. 1g). Additional qualitative histological and immunohistochemical analyses revealed markedly increased paw swelling and dilated lymphatic vessels on day 3, reflecting acute lymph stasis (Fig. 1h,j). In contrast, 10 days after surgery, paw volume had decreased and lymphatic vessels exhibited normal configuration (Fig. 1i,k).

### 3D hindlimb volumetry

**Figure 2:**
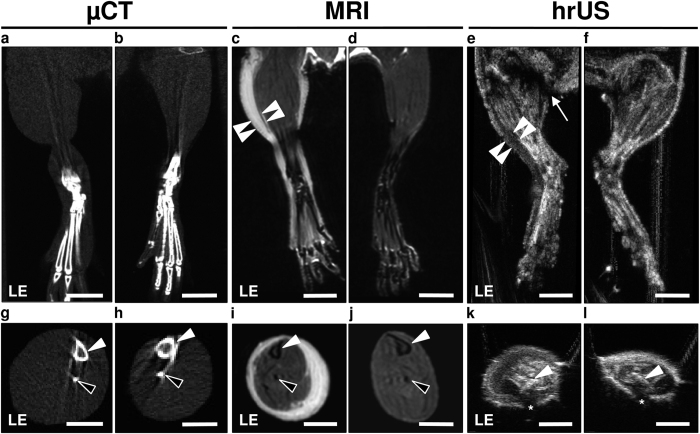
µCT, MRI and hrUS for hindlimb volumetry. (**a–l**) Coronal (**a–f**) and axial (**g–l**) hindlimb images of μCT (**a,b,g**,**h**), MRI (**c,d,i**,**j**) and hrUS (**e,f,k**,**l**) 3 days after popliteal lymphadenectomy. In T2-weighted MR images, the thickened and edematous tissue was characterized by epifascial hyperintensity (**c**, arrowheads). In contrast, 30 MHz hrUS objectified lymphedema as a hypoechoic layer (**e**, arrowheads). (**e**) Arrow = gluteal fold, which was used as a landmark for volume calculation. In axial images, tibia (**g–l**, white arrowhead) and fibula (**g–j**, black arrowheads) can be reliably identified. Note the dorsal acoustic attenuation in hrUS (**k**,**l**, asterisk). LE = lymphedema; scales (**a–f)** = 4.5 mm; (**g–l)** = 3 mm.

The volumes of operated and contralateral non-operated hindlimbs were assessed by means of μCT, 9.4 T MRI and hrUS (Fig. 2a–l). The analysis included the determination of the overall volume (in mm^3^) of each hindlimb. For this purpose, boundaries were manually outlined in parallel slices separated by 1 mm step size in the 3D modality images and volumes were calculated by integrating the outlined areas. Volumes were assessed with the gluteal skinfold as a landmark (Fig. 2e), because circumferential hindlimb boundaries could be outlined up to this point.

All 3D techniques resulted in high-resolution hindlimb images. In addition, due to high soft-tissue contrast, MRI and hrUS allowed visualization of edematous and thickened dermal tissue in operated hindlimbs (Fig. 2c,e,i,k). Volume assessment with hrUS was complicated by dorsal acoustic attenuation in axial images, which was overcome by extrapolation when measuring limb circumference (Fig. 2k,l). Examination times for the different 3D techniques are summarized in Table 1.

**Figure 3:**
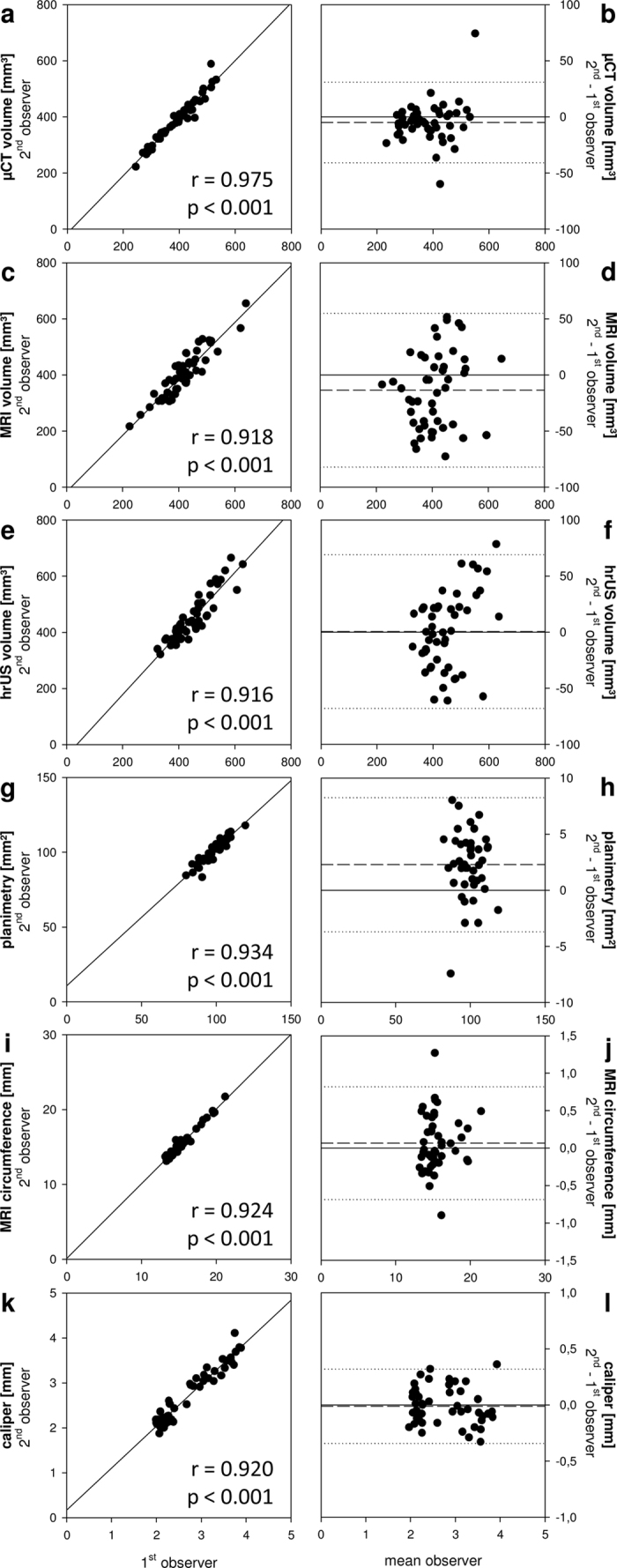
Interobserver variability of hindlimb volumetry modalities. (**a–l**) μCT showed the highest correlation between two observers (**a**) and a low measurement variability (**b**) compared to other modalities as assessed by linear correlation (**a,c,e,g,i**,**k**) and Bland-Altman analyses (**b,d,f,h,j**,**l**). Despite low interobserver variability, MRI, hrUS, planimetry and MRI-measured circumference were associated with higher measurement variations (**c–j**). Dashed line = mean difference between observers; dotted line = double standard deviation. n = 48 for μCT, MRI, hrUS, MRI circumference and caliper; n = 42 for planimetry.

**Figure 4:**
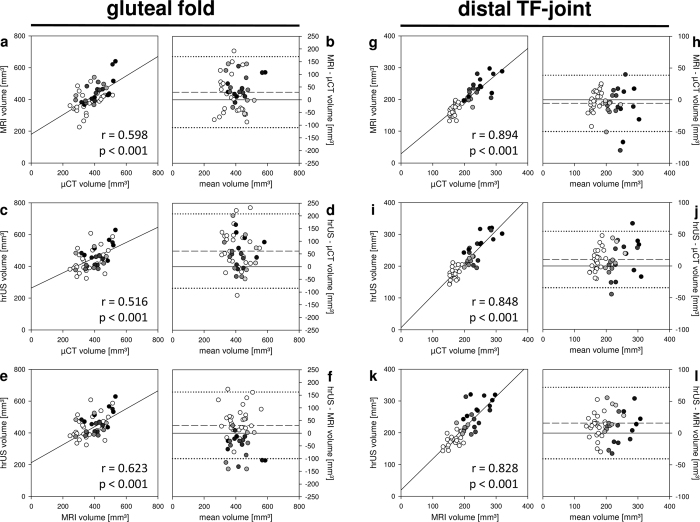
Correlation between different 3D volumetry modalities. (**a–f**) Linear correlations (**a,c**,**e**) and Bland-Altman plots (**b,d**,**f**) of 3D volumetry using the gluteal fold as landmark. Hindlimb volumes are illustrated in grey scale with non-operated controls (white circles) and operated limbs (day 3–10: black to bright grey circles). The volumes calculated with the gluteal fold landmark correlated poorly among the 3D modalities and led to a random distribution of the volumes without the expected grouping into control and operated hindlimb volumes. (**g–l**) After standardized measuring with the distal TF-joint landmark, 3D volumetry exhibited good correlations (**g,i**,**k**). However, the higher the hindlimb volumes, the higher the measurement variability was as shown in Bland-Altman analyses (**h,j**,**l**; black circles). Dashed line = mean difference between volume measurements; dotted line = double standard deviation; n = 48.

**Figure 5:**
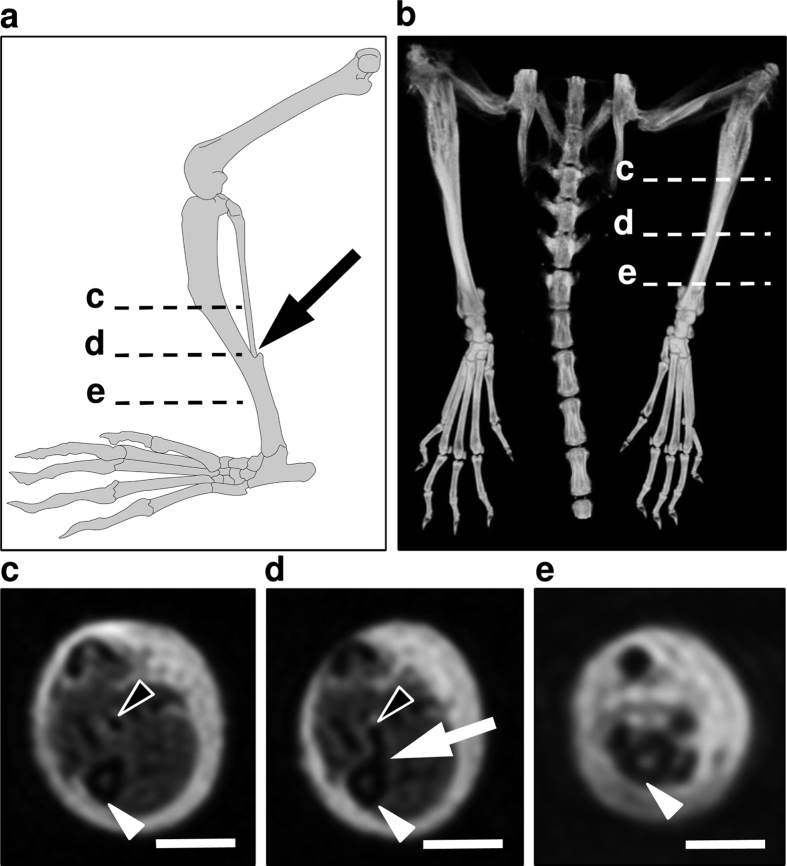
The distal TF-joint as landmark for standardized 3D limb volumetry. (**a–e**) The distal TF-joint was located in the distal third of the mouse hindlimb, as shown in an illustration (**a**) and 3D reconstruction of the hindlimb skeleton (**b**). It could be reliably localized in axial images of 3D modalities (**c,d**,**e**). Proximal to the TF-joint (**c**), both tibia (white arrowhead) and fibula (black arrowhead) were detectable. More distally, the two bones articulated (**d**, arrow). The level of the TF-joint was used for MRI-based calculations of limb circumference. (**a**) was drawn by Carol De Simio (University Hospital Zurich).

Using the gluteal skinfold as a landmark, the linear correlation between volume measurements of two observers was high for all 3D techniques (Fig. 3a,c,e). μCT 3D volumetry showed the highest correlation with r = 0.975 (Fig. 3a,b). In contrast, MRI and hrUS showed a higher measurement variability between observers as illustrated by additional Bland-Altman analyses (Fig. 3d,f). Moreover, there was an insufficient correlation among 3D volumetric modalities (Fig. 4a–f). To overcome this inaccuracy, we developed a more standardized method to calculate hindlimb volumes in 3D modalities. For this purpose, the distal tibio-fibular (TF) joint was defined as a new landmark and the limb volume distal to the joint was calculated (Fig. 5). Volumes calculated using the distal TF-joint landmark correlated well among the 3D techniques (Fig. 4g–l).

**Figure 6:**
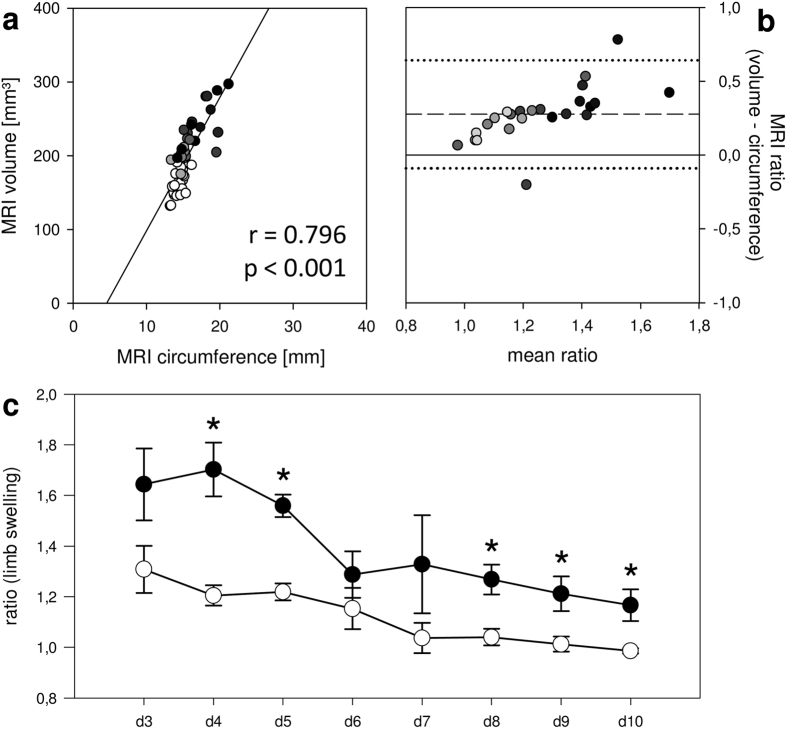
3D volume and circumference measurements with MRI. (**a–c**) The two types of measurement correlated (**a**) but 3D volumetry revealed markedly higher limb ratios, as depicted by Bland-Altman analysis (**b**). Dashed line = mean difference between ratios; dotted line = double standard deviation; n = 48; day 3-10 = black to bright grey circles; non-operated control limbs = white circles. **(c)** Limb ratios based on 3D volumes were consistently higher throughout the course of the study (3D volume: black circles; circumference: white circles; n = 3 for each time point; mean ± SEM; *p < 0.05).

The measurement of limb circumference has been frequently used to monitor experimental lymphedema in rodents. However, measuring with a string is prone to inaccuracy due to the small limb and the risk of compression. To prevent methodological inaccuracies of this conventional technique, we evaluated limb circumferences in axial T2-weighted MR images at the distal TF joint (steric trapping occurs within 5 minutes (Fig. 2b and Fig. S1a,b). This approach also allowed an accurate comparison of MRI-based limb circumferences with 3D volumes. The correlation between the two modalities was acceptable (r = 0.796; Fig. 6a). However, the calculation of hindlimb ratios (operated divided by non-operated leg) showed that circumferential measuring was less sensitive for the detection of lymphedema than 3D volumetry (Fig. 6b,c).

### Conventional measuring techniques

The volumes of operated and contralateral non-operated hindlimbs were additionally assessed by means of planimetry, circumferential length and paw thickness measurements. For planimetric analyses, photographs of the operated and non-operated limbs were taken under a stereomicroscope and recorded on DVD. The images were analyzed by means of the software package ImageJ^23^. For this purpose, we measured the limb area in a standardized template (Fig. S1c,d). Paw thickness was measured with an electronic caliper. To standardize measurements, they were performed in a transverse technique between the first and second proximal pad of the paw for all time points (Fig. S1e).

**Figure 7:**
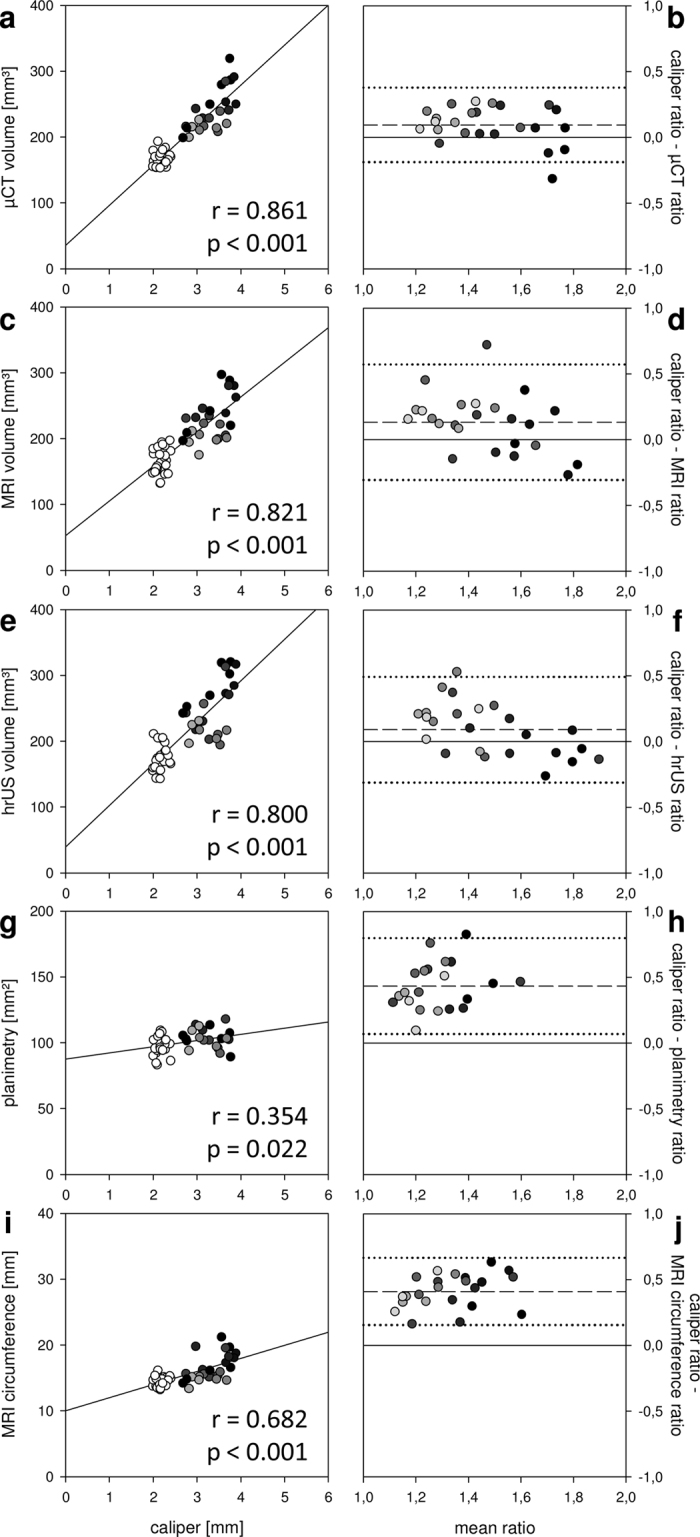
Caliper-measured paw thickness versus other hindlimb volumetry modalities. (**a–j**) Linear correlations (**a,c,e,g**,**i**) and Bland-Altman plots (**b,d,f,h**,**j**) comparing caliper-measured paw thickness with other hindlimb volumetry modalities. The caliper correlated well with 3D volumetry (**a–f**) but no or low correlation with planimetry (**g,h**) and circumferential length (**i,j**) was recorded. Importantly, hindlimb ratios based on paw thickness were higher than those calculated with the other modalities (n = 48 for μCT, MRI, hrUS, MRI circumference and caliper; n = 42 for planimetry; day 3–10 = black to bright grey circles; non-operated control limbs = white circles; dashed line = mean difference between ratios; dotted line = double standard deviation).

We found high interobserver correlations of all conventional measuring techniques (Fig. 3g,i,k). Caliper measurements revealed fewer outliers than the other approaches. Importantly, paw thickness correlated well with 3D volumes measured by μCT, MRI or hrUS (Fig. 7a–f). Surprisingly, there was no correlation with two-dimensional planimetry (Fig. 7g,h) and only moderate correlation with circumferential length measurement (Fig. 7i,j). Additionally, the assessment of paw thickness resulted in higher leg ratios than the other techniques (Fig. 7b,d,f,h,j).

## Discussion

The high prevalence of cancer-related lymphedema indicates urgent needs for novel treatment strategies. Microsurgical interventions to treat lymphedema include the transplantation of lymphatic vessels^24^, lymphatico-venular anastomosis^25^ or vascularized lymph node transfer^26^. These approaches do not reverse the underlying pathophysiology and may only provide stabilization or delay in the development of end-stage sequelae as disfigurement and loss of function^5^.

Robust animal models, such as the rodent hindlimb, are indispensable for the establishment of novel treatments. However, beside translational issues, the lack of knowledge about the quality of volumetric modalities is a major concern in experimental lymphology^11^. Therefore, in the present study we assessed in detail the performance of different volumetric techniques for the mouse hindlimb.

Surgery in combination with irradiation is commonly applied for the induction of chronic lymphedema in animals^7^,^22^. Nonetheless, approaches inducing acute lymphatic damage without irradiation have been used for the study of vascularized lymph node transfer and growth factor treatment^27^,^28^. In addition, Mendez *et al*.^13^ demonstrated in an acute lymphedema model of the rat forelimb that removal of axillary lymph nodes induces a chronic and latent lymphatic insufficiency. Herein, we used such a modified acute lymphedema model for the analysis of limb volumetry. The combination of radical popliteal lymphadenectomy, circular skin incision and cautery led to limb swelling which persisted up to 28 days. The rapid decrease of postsurgical hindlimb swelling was particularly useful for correlation analyses of different volumetry techniques due to a well-distributed data set.

Recently, 3D imaging modalities have been applied for limb volumetry in humans^29^ and rodents^21^,^22^ with lymphedema. Little is known about their validity in comparison to conventional methods such as the assessment of circumferential length or paw thickness. Therefore, we analyzed μCT-, MRI- and hrUS-volumetry in the mouse hindlimb. All modalities were characterized by a high interobserver correlation. We further found that high correlations between the different 3D techniques crucially depend on standardized landmarks for volume calculation. The assessment of hindlimb volumes using the gluteal fold as a landmark was inaccurate, probably due to variable positioning of the animals. In contrast, volume calculations using a more reproducible landmark, i.e. the distal TF-joint, yielded high correlation coefficients. These novel findings suggest that it may be reasonable to limit experimental 3D limb volumetry to distal parts of the extremity, as more proximal areas are hardly assessable without positioning-dependent measurement errors.

In contrast to conventional techniques, 3D volumetry modalities allow the visualization of histopathological lymphedema hallmarks such as soft tissue fibrosis and fat deposition. Moreover, they enable dynamic imaging of the lymphatic system. However, in preclinical research with small rodents, intralymphatic application of contrast agents is nearly impossible^30^. In close analogy to human procedures, indirect MR lymphangiography with intradermal contrast application has been employed for dynamic imaging of the lymphatic system in mice^31^,^32^,^33^. CT-based lymphangiography similarly depends on the development of lymph-affine contrast agents and promising advances have been made in the field^34^. While μCT and MRI are more established, photo-acoustic detection of lymphatic vessels is still in its infancy^35^. Because most conventional contrast agents are not specifically absorbed by the lymphatic system and, thus, are not suitable for small animal lymphangiography, a broad preclinical use of MRI and μCT for this purpose is still restricted to expensive or not commercially available products.

Many researches use conventional techniques to estimate the rodent hindlimb volume when reporting outcomes of experimental lymphedema treatment. Common parameters are the assessment of water displacement^36^, circumferential length^37^,^38^,^39^ or paw thickness^7^,^19^. Importantly, while well established in human lymphology^40^, water displacement has been shown to yield inaccurate measurements in the mouse-tail model^41^. Measuring hindlimb volumes with water displacement is even more challenging due to the small and short extremities and difficult standardization. Therefore, unless plethysmometers are available^36^,^42^, water displacement may be inaccurate for the assessment of rodent limb volumes. Accordingly, we did not perform water displacement in this study.

The caliper represents an inexpensive and simple tool for the assessment of rodent paw thickness. It has been used as a surrogate parameter for hindlimb volume in experimental lymphedema research^7^,^19^, but its eligibility for this purpose has not been specifically analyzed. Planimetry as well as the measurement of circumferential length and paw thickness showed high interobserver correlations. Moreover, caliper-measured paw thickness was characterized by the smallest measurement variation even though it revealed the highest values for limb swelling as assessed by means of leg ratio. The correlation of the caliper measurements with all 3D volumetry modalities was high (r = >0.8) and Bland-Altman analyses revealed that caliper-measured leg ratios were slightly higher than those of μCT, MRI and hrUS. Interestingly, planimetry and circumferential length poorly correlated with paw thickness. The question, whether the caliper overestimates the ratio, i.e. the severity of lymphedema, or whether other modalities tend to underestimate the volume deserves special attention. In fact, gravitational forces influence lymphedema in humans^43^, a phenomenon that could also aggravate rodent paw swelling. Therefore, the caliper may be especially sensitive for the assessment of rodent limb lymphedema. Furthermore, paw thickness is already a well-established parameter in experimental research of inflammatory arthritis^44^,^45^.

Taken together, this study demonstrates that the calculation of hindlimb volumes in mice can be achieved in different ways. 3D techniques such as μCT, MRI and hrUS are expensive, time-consuming and must be strictly standardized for valid and reliable measurements. In contrast, caliper-measured paw thickness may be the most suitable method to assess the course of rodent lymphedema. It represents an inexpensive, technically feasible, fast and reproducible method with a high sensitivity to detect changes of paw volume.

## Methods

### Animals

For the establishment of the acute lymphedema model, we used male C57BL/6 mice (Institute for Clinical & Experimental Surgery, Saarland University, Homburg/Saar, Germany) with a body weight of 28–31 g (n = 6). For the volumetric study, male C57BL/6 mice with a body weight of 25–27 g (n = 12) were used. The animals were housed one per cage with a 12-h day/night cycle and were fed ad libitum with water and standard pellet food (Atromin, Lage, Germany). The local governmental animal care committee (Landesamt für Verbraucherschutz, Saarbrücken, Germany) approved all experiments. They were conducted in accordance with the European legislation on the protection of animals (Directive 2010/63/EU) and the NIH guidelines on the care and use of laboratory animals (NIH publication #85-23 Rev. 1985).

### Animal model

Mice were anesthetized by intraperitoneal injection of ketamine (75 mg/kg body weight; Ursotamin, Serumwerk Bernburg AG, Bernburg, Germany) and xylazine (15 mg/kg body weight; Rompun, Bayer, Leverkusen, Germany). To induce lymphedema, we combined and modified the surgical steps of previously established hindlimb models^19^,^38^. For this purpose, hindlimbs were depilated (Nair hair removal cream, Church & Dwight Canada Corp., Mississauga, ON, Canada) and the animals were placed in prone position. After intradermal injection of 15 μL methylene blue 10% (Carl Roth GmbH, Karlsruhe, Germany) in the right middle phalanx with a 31 Gauge syringe (Hamilton Bonaduz AG, Bonaduz, Switzerland) (Fig. 1a), a circular incision was performed over the popliteal fossa. The stained afferent lymphatic vessels were ligated with 10/0 monofilament (Monosof; Covidien Deutschland GmbH, Neustadt/Donau, Germany) and the fat pad containing the popliteal lymph node and the efferent lymphatic vessels was microsurgically resected under continuous hemostasis (Erbe Kauter B; Erbe Elektromedizin GmbH, Tübingen, Germany) maintaining the surrounding muscles intact (Fig. 1b,c). Finally, circumferential epifascial cautery was performed to block superficial lymphatic vessels. The skin was closed with interrupted 5/0 monofilament (Prolene; Ethicon, Johnson & Johnson Medical GmbH, Norderstedt, Germany). Postoperative analgesia was provided for 3 days with tramalhydrochloride (40 mg/100 mL drinking water; Grünenthal GmbH, Aachen, Germany). The contralateral hindlimb was not operated and served as internal control.

### μCT

Imaging was performed in isoflurane anesthesia and supine position by means of the *in vivo* SkyScan 1076 μCT system (Bruker, Kontich, Belgium). The 35 mm transaxial field of view (FOV) allowed imaging of both hindlimbs. An oversize scan, which was performed by connecting 3 scans with an estimated acquisition time of 11 min, enabled the visualization of the hindlimbs from phalanx to pelvis. The scan parameters were 35 μm pixel size, 50 kV, 200 μA, 0.5 mm Al filter, angular rotation step 0.8° and an exposure time of 58 ms. The recorded images were segmented using an adaptive thresholding algorithm provided by the SkyScan software CTan, because the observation of bone and soft tissue precluded the use of global thresholding^46^. Quantitative analysis of the 3D measurements was performed by means of the appropriate software licensed to Bruker (CTan).

### MRI

MRI was performed in isoflurane anesthesia using a linear polarized coil (inner diameter: 38 mm) developed for imaging of the mouse abdomen and a horizontal bore 9.4 T MRI animal scanner equipped with the operating software Paravision 6.0.1 (Bruker Biospin Inc., Billerica, MA, USA) (Fig. S1f). Because hindlimb imaging was prone to partial volume effects, we used a water-filled phantom to facilitate sufficient magnetic field homogeneity.

The hindlimb protocol consisted of a fast low angle shot based 3D localizer, followed by extensive 1^st^ and 2^nd^ order shimming of the entire FOV. Subsequently, adjustments of resonance frequency, radio frequency pulse strength and receiver gain were performed and slice geometry was adjusted to animal placement within the isocenter. 3D volumetry datasets were recorded with a 3D rapid acquisition relaxation enhanced (RARE) sequence with flipback RF pulse, facilitating T2-weighted imaging with low repetition time (TR). Datasets were recorded with the following settings: Slice orientation coronal, TR 500 ms, echo time 33 ms, RARE factor 16, excitation/re-focussing flip angle 90°/180°, number of averages 2, zero fill in read phase and slice directions 2, FOV 42 × 35 × 20 mm^3^, matrix size 420 × 350 × 200, resulting voxel size 100 × 100 × 100 μm^3^. Axial MR images were reconstructed with the MRI scanner operating software and exported as DICOM (Digital Imaging and Communications in Medicine) images for further processing with the free DICOM software OsiriX™ Lite (version v.7.0.3; Pixmeo, Geneva, Switzerland). The hindlimb volumes were manually calculated by integrating the outlined areas for MRI measurements using a spreadsheet program (Office Excel^®^ 2007; Microsoft, Redmond, WA, USA).

### hrUS

Mice were anesthetized with isoflurane and put on a heated stage (Fig. S1g). Chemical depilation (Nair hair removal cream) was done to prevent air trapped in the fur from interfering with ultrasound coupling into the animal. To distinguish between the stage and the hindlimb, ultrasound coupling gel (Aquasonic 100, Parker, Fairfield, NJ, USA) was circumferentially applied to hindlimbs and pelvis (Fig. S1g).

Imaging was performed by means of a Vevo 770 high-resolution ultrasound system (VisualSonics, Toronto, ON, Canada) and a real-time microvisualization 707B Scanhead (VisualSonics) with a center frequency of 30 MHz and a focal depth of 12.5 mm. For 3D imaging the scanhead, driven by a linear motor, scanned first the non-operated and then the operated hindlimb. The technique yielded two-dimensional (2D) images at parallel and uniformly spaced 100 μm steps. The 2D image planes enabled rapid 3D image reconstruction, displaying a dynamic cube view format, as previously described^47^. Volume calculations were performed with the software licensed VisualSonics (Vevo 770 V2.3.0).

### Histology and immunohistochemistry

For light microscopy, formalin-fixed paws were embedded in paraffin. The paraffin blocks were cut until the desired plane and decalcified with Osteomoll^®^ (Merck KGaA, Darmstadt, Germany) for 10 min. Subsequently, 2 μm sections were cut and stained with hematoxylin and eosin (HE) according to standard procedures. For the immunohistochemical detection of lymphatic vessels, additional sections were stained with a polyclonal rabbit IgG-antibody against lymphatic vessel endothelial hyaluronan receptor (LYVE)-1 (1:50; Abcam, Cambridge, UK). A peroxidase-labeled goat-anti-rabbit IgG-antibody (1:200; Dianova, Hamburg, Germany) served as secondary antibody and 3-amino-9-ethylcarbazole (ready-to-use, ab64252; Abcam) was added as chromophor. The sections were counterstained with Mayer’s hemalum solution (HX948000; Merck, Darmstadt, Germany) and examined under a BX60 microscope (Olympus, Hamburg, Germany) for qualitative analyses.

### Statistics

Data were analyzed for normal distribution and equal variance. Differences between non-operated and operated legs were tested by an unpaired Student’s t test. Linear correlation (Pearson’s coefficient of correlation for parametric distribution or Spearman’s coefficient of correlation for nonparametric distribution) and Bland-Altman analyses were performed to evaluate correspondence of different measurement techniques detecting hindlimb swelling. To create a Bland-Altman plot, the mean of all measured values detected by two different observers or techniques was plotted on the horizontal axis and the difference between the two types of measurement was plotted on the vertical axis^48^. Values were expressed as mean ± standard error of the mean (SEM). Statistical significance was set for p < 0.05.

## Additional Information

**How to cite this article**: Frueh, F. S. *et al*. High-resolution 3D volumetry versus conventional measuring techniques for the assessment of experimental lymphedema in the mouse hindlimb. *Sci. Rep.* **6**, 34673; doi: 10.1038/srep34673 (2016).

## Supplementary Material

Supplementary Information

## Figures and Tables

**Table 1:** Features of different techniques for rodent hindlimb volumetry.

Modality	Costs	Examination time (min ± SD)	Anesthesia required?	Lymphatic imaging possible?
μCT	+ + +	14.6 ± 2.2	yes	yes
MRI	+ + +	34.9 ± 2.8	yes	yes
hrUS	+ + +	4.9 ± 0.9	yes	no (yes)
Planimetry	+ +	1.0 ± 0.3	yes	no
Circumference	+	not assessed	no	no
Caliper	+	1.2 ± 0.2	no	no

3D techniques are associated with high costs, longer examination times (i.e. time span from anesthetic induction to end of imaging) and always require anesthesia. They bear the advantage of additional lymphatic imaging using lymph-affine contrast agents. In contrast, conventional techniques are time- and cost-efficient methods to assess surrogate parameters for rodent hindlimb volumes. For maximal accuracy, hindlimb planimetry should be performed under anesthesia as well.

## References

[b1] Cueni L. N. & Detmar M. The lymphatic system in health and disease. Lymphat. Res. Biol. 6, 109–122 (2008). 19093783 10.1089/lrb.2008.1008 PMC3572233

[b2] Rockson S. G. Current concepts and future directions in the diagnosis and management of lymphatic vascular disease. Vasc. Med. 15, 223–231 (2010). 20483987 10.1177/1358863X10364553

[b3] Szuba A. & Rockson S. G. Lymphedema: anatomy, physiology and pathogenesis. Vasc. Med. 2, 321–326 (1997). 9575606 10.1177/1358863X9700200408

[b4] Alitalo K. The lymphatic vasculature in disease. Nat. Med. 17, 1371–1380 (2011). 22064427 10.1038/nm.2545

[b5] Mortimer P. S. & Rockson S. G. New developments in clinical aspects of lymphatic disease. J. Clin. Invest. 124, 915–921 (2014). 24590276 10.1172/JCI71608 PMC3938261

[b6] Karkkainen M. J. . Missense mutations interfere with VEGFR-3 signalling in primary lymphoedema. Nat. Genet. 25, 153–159 (2000). 10835628 10.1038/75997

[b7] Bramos A. . Prevention of Postsurgical Lymphedema by 9-cis Retinoic Acid. Ann. Surg. 264, 353–361 (2016). 26655920 10.1097/SLA.0000000000001525 PMC7513676

[b8] Avraham T. . Fibrosis is a key inhibitor of lymphatic regeneration. Plast. Reconstr. Surg. 124, 438–450 (2009). 19644258 10.1097/PRS.0b013e3181adcf4b

[b9] Hadamitzky C. & Pabst R. Acquired lymphedema: an urgent need for adequate animal models. Cancer Res. 68, 343–345 (2008). 18199525 10.1158/0008-5472.CAN-07-2454

[b10] DiSipio T., Rye S., Newman B. & Hayes S. Incidence of unilateral arm lymphoedema after breast cancer: a systematic review and meta-analysis. Lancet Oncol. 14, 500–515 (2013). 23540561 10.1016/S1470-2045(13)70076-7

[b11] Frueh F. S. . Animal models in surgical lymphedema research--a systematic review. J. Surg. Res. 200, 208–220 (2016). 26235906 10.1016/j.jss.2015.07.005

[b12] Mendez U., Brown E. M., Ongstad E. L., Slis J. R. & Goldman J. Functional recovery of fluid drainage precedes lymphangiogenesis in acute murine foreleg lymphedema. Am. J. Physiol. Heart. Circ. Physiol. 302, H2250–H2256 (2012). 22427513 10.1152/ajpheart.01159.2011 PMC3378291

[b13] Mendez U., Stroup E. M., Lynch L. L., Waller A. B. & Goldman J. A chronic and latent lymphatic insufficiency follows recovery from acute lymphedema in the rat foreleg. Am. J. Physiol. Heart. Circ. Physiol. 303, H1107–H1113 (2012). 22942182 10.1152/ajpheart.00522.2012 PMC3517646

[b14] Tammela T. . Therapeutic differentiation and maturation of lymphatic vessels after lymph node dissection and transplantation. Nat. Med. 13, 1458–1466 (2007). 18059280 10.1038/nm1689

[b15] Aschen S. Z. . Lymph node transplantation results in spontaneous lymphatic reconnection and restoration of lymphatic flow. Plast. Reconstr. Surg. 133, 301–310 (2014). 24469165 10.1097/01.prs.0000436840.69752.7e PMC4066306

[b16] Cheng M. H. . The mechanism of vascularized lymph node transfer for lymphedema: natural lymphaticovenous drainage. Plast. Reconstr. Surg. 133, 192e–198e (2014). 10.1097/01.prs.0000437257.78327.5b 24469190

[b17] Joseph W. J. . Sterile inflammation after lymph node transfer improves lymphatic function and regeneration. Plast. Reconstr. Surg. 134, 60–68 (2014). 25028818 10.1097/PRS.0000000000000286 PMC4101920

[b18] Nguyen D. H. . Quantity of lymph nodes correlates with improvement in lymphatic drainage in treatment of hind limb lymphedema with lymph node flap transfer in rats. Microsurgery. 36, 239–245 (2016). 25715830 10.1002/micr.22388

[b19] Hwang J. H. . Therapeutic lymphangiogenesis using stem cell and VEGF-C hydrogel. Biomaterials. 32, 4415–4423 (2011). 21421266 10.1016/j.biomaterials.2011.02.051

[b20] Yoshida S. . Adipose-derived stem cell transplantation for therapeutic lymphangiogenesis in a mouse secondary lymphedema model. Regen. Med. 10, 549–562 (2015). 26237700 10.2217/rme.15.24

[b21] Sommer T. . Quantification of lymphedema in a rat model by 3D-active contour segmentation by magnetic resonance imaging. Lymphat. Res. Biol. 10, 25–29 (2012). 22416909 10.1089/lrb.2011.0010

[b22] Yang C. Y. . Developing a Lower Limb Lymphedema Animal Model with Combined Lymphadenectomy and Low-dose Radiation. Plast. Reconstr. Surg. Glob. Open. 2, e121 (2014). 25289315 10.1097/GOX.0000000000000064 PMC4174147

[b23] Schneider C. A., Rasband W. S. & Eliceiri K. W. NIH Image to ImageJ: 25 years of image analysis. Nat. Methods. 9, 671–675 (2012). 22930834 10.1038/nmeth.2089 PMC5554542

[b24] Baumeister R. G. . Microsurgical Lymphatic Vessel Transplantation. J. Reconstr. Microsurg. 32, 34–41 (2016). 26165882 10.1055/s-0035-1554934

[b25] Koshima I., Inagawa K., Urushibara K. & Moriguchi T. Supermicrosurgical lymphaticovenular anastomosis for the treatment of lymphedema in the upper extremities. J. Reconstr. Microsurg. 16, 437–442 (2000). 10993089 10.1055/s-2006-947150

[b26] Becker C., Assouad J., Riquet M. & Hidden G. Postmastectomy lymphedema: long-term results following microsurgical lymph node transplantation. Ann. Surg. 243, 313–315 (2006). 16495693 10.1097/01.sla.0000201258.10304.16 PMC1448940

[b27] Lähteenvuo M. . Growth factor therapy and autologous lymph node transfer in lymphedema. Circulation. 123, 613–620 (2011). 21282502 10.1161/CIRCULATIONAHA.110.965384

[b28] Honkonen K. M. . Lymph node transfer and perinodal lymphatic growth factor treatment for lymphedema. Ann. Surg. 257, 961–967 (2013). 23013803 10.1097/SLA.0b013e31826ed043

[b29] Hwang J. H., Lee C. H., Lee H. H. & Kim S. Y. A new soft tissue volume measurement strategy using ultrasonography. Lymphat. Res. Biol. 12, 89–94 (2014). 24521479 10.1089/lrb.2013.0030 PMC4062108

[b30] Sevick-Muraca E. M., Kwon S. & Rasmussen J. C. Emerging lymphatic imaging technologies for mouse and man. J. Clin. Invest. 124, 905–914 (2014). 24590275 10.1172/JCI71612 PMC3938259

[b31] Kobayashi H. . Comparison of dendrimer-based macromolecular contrast agents for dynamic micro-magnetic resonance lymphangiography. Magn. Reson. Med. 50, 758–766 (2003). 14523962 10.1002/mrm.10583

[b32] Pan D., Suzuki Y., Yang P. C. & Rockson S. G. Indirect magnetic resonance lymphangiography to assess lymphatic function in experimental murine lymphedema. Lymphat. Res. Biol. 4, 211–216 (2006). 17394404 10.1089/lrb.2006.4405

[b33] Mounzer R. . Dynamic imaging of lymphatic vessels and lymph nodes using a bimodal nanoparticulate contrast agent. Lymphat. Res. Biol. 5, 151–158 (2007). 18035933 10.1089/lrb.2007.5302

[b34] Hayashi K., Nakamura M. & Ishimura K. Near-infrared fluorescent silica-coated gold nanoparticle clusters for x-ray computed tomography/optical dual modal imaging of the lymphatic system. Adv. Healthc. Mater. 2, 756–763 (2013). 23184510 10.1002/adhm.201200238

[b35] Martel C. . Photoacoustic lymphatic imaging with high spatial-temporal resolution. J. Biomed. Opt. 19, 116009 (2014). 25408958 10.1117/1.JBO.19.11.116009 PMC4407768

[b36] Shejawal N., Menon S. & Shailajan S. A simple, sensitive and accurate method for rat paw volume measurement and its expediency in preclinical animal studies. Hum. Exp. Toxicol. 33, 123–129 (2014). 23584356 10.1177/0960327113482594

[b37] Lee-Donaldson L. . Refinement of a rodent model of peripheral lymphedema. Lymphology. 32, 111–117 (1999). 10494523

[b38] Oashi K. . A new model of acquired lymphedema in the mouse hind limb: a preliminary report. Ann. Plast. Surg. 69, 565–568 (2012). 21629054 10.1097/SAP.0b013e31821ee3dd

[b39] Park H. S. . Modification of a rodent hindlimb model of secondary lymphedema: surgical radicality versus radiotherapeutic ablation. Biomed. Res. Int. 2013, 208912 (2013). 24350251 10.1155/2013/208912 PMC3856125

[b40] Damstra R. J., Glazenburg E. J. & Hop W. C. Validation of the inverse water volumetry method: A new gold standard for arm volume measurements. Breast Cancer Res. Treat. 99, 267–273 (2006). 16752072 10.1007/s10549-006-9213-0

[b41] Pan D., Han J., Wilburn P. & Rockson, Validation of a new technique for the quantitation of edema in the experimental setting. Lymphat. Res. Biol. 4, 153–158 (2006). 17034295 10.1089/lrb.2006.4.153

[b42] Shioya R. . Prevention of Lymphedematous Change in the Mouse Hindlimb by Nonvascularized Lymph Node Transplantation. Ann. Plast. Surg. 76, 442–445 (2016). 25664410 10.1097/SAP.0000000000000428

[b43] Fajardo K. A. . Bilateral lower extremity inflammatory lymphedema in Air Force basic trainees: clinical and epidemiologic study of a new disease entity. JAMA Dermatol. 151, 395–400 (2015). 25607253 10.1001/jamadermatol.2014.3794

[b44] Zhou H. F., Chan H. W., Wickline S. A., Lanza G. M. & Pham C. T. Alphavbeta3-targeted nanotherapy suppresses inflammatory arthritis in mice. FASEB J. 23, 2978–2985 (2009). 19376816 10.1096/fj.09-129874 PMC2735365

[b45] Xin W. . Methyl salicylate lactoside inhibits inflammatory response of fibroblast-like synoviocytes and joint destruction in collagen-induced arthritis in mice. Br. J. Pharmacol. 171, 3526–3538 (2014). 24712652 10.1111/bph.12715 PMC4105938

[b46] Laperre K. . Development of micro-CT protocols for *in vivo* follow-up of mouse bone architecture without major radiation side effects. Bone. 49, 613–622 (2011). 21763477 10.1016/j.bone.2011.06.031

[b47] Fenster A., Downey D. B. & Cardinal H. N. Three-dimensional ultrasound imaging. Phys. Med. Biol. 46, R67–R99 (2001). 11384074 10.1088/0031-9155/46/5/201

[b48] Bland J. M. & Altman D. G. Statistical methods for assessing agreement between two methods of clinical measurement. Lancet. 1, 307–310 (1986). 2868172

