# Supplementary material for: High-resolution 3D volumetry versus conventional measuring techniques for the assessment of experimental lymphedema in the mouse hindlimb
Source: Sci Rep. 2016 Oct 4;6:34673. doi: 10.1038/srep34673 (PMC5048170; doi:10.1038/srep34673)
Supplement: Supplementary Information [file srep34673-s1.pdf]

Supplementary Information

High-resolution 3D volumetry versus conventional measuring techniques for the assessment of experimental lymphedema in the mouse hindlimb

Florian S. Frueh[1,4,§,*], Christina Körbel[1,§], Laura Gassert[1], Andreas Müller[2], Epameinondas Gousopoulos[3], Nicole Lindenblatt[4], Pietro Giovanoli[4], Matthias W. Laschke[1], Michael D. Menger[1]

[§] Equal contributors

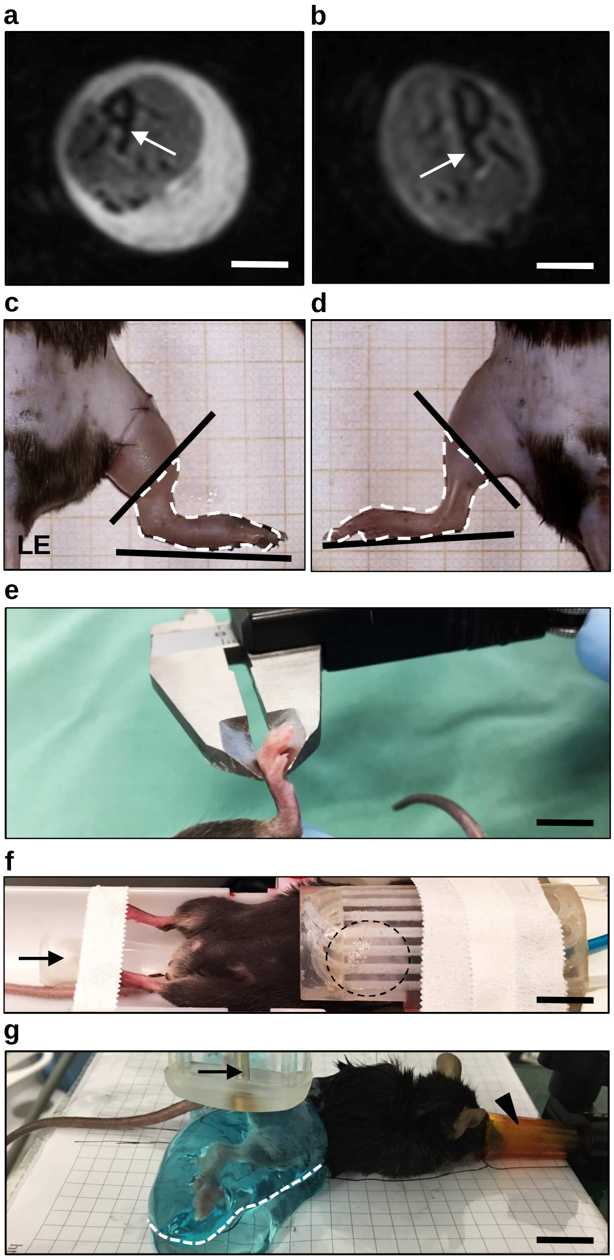

Figure S1. Techniques for murine hindlimb volumetry. (**a, b**) Axial T2-weighted MR images of an operated (**a**) and non-operated (**b**) limb at day 3. The assessment of circumferential length was performed under standardized conditions at the distal tibio-fibular joint (arrows). Scales = 1.8 mm. (**c, d**) Planimetric assessment of hindlimb area. The analysis was performed in a standardized template (borders marked by black lines). (**e**) Paw thickness was measured using an electronic caliper. Scale = 7mm. (**f**) MRI with a 9.4 T scanner. The animals were placed in supine and tail-first position. Dotted circle = sensor for respiratory rate monitoring; arrow = water-filled phantom. For µCT imaging, the animals were similarly

positioned. Scale = 10mm. (**g**) For hrUS, the animals were placed in prone position. An outline of a mouse body on top of the heated stage guaranteed equal fixation of each mouse. Arrow: scanhead. Arrowhead: isoflurane-mask. Scale = 12mm.
